# Surface Functionalisation of Hyaluronic Acid-Based Foams with TiO_2_ via ALD: Structural, Wettability and Antimicrobial Properties Analysis for Biomedical Applications

**DOI:** 10.3390/ma18245530

**Published:** 2025-12-09

**Authors:** Ewelina Pabjańczyk-Wlazło, Nina Tarzyńska, Anna Bednarowicz, Adam K. Puszkarz, Grzegorz Szparaga, Sławomir Sztajnowski, Piotr Kaczmarek

**Affiliations:** 1 Textile Institute, Lodz University of Technology, 116 Zeromskiego Street, 90-924 Lodz, Poland; adam.puszkarz@p.lodz.pl (A.K.P.); grzegorz.szparaga@p.lodz.pl (G.S.); slawomir.sztajnowski@p.lodz.pl (S.S.); 2 Centre for Biomedical Engineering, ŁUKASIEWICZ Research Network—Lodz Institute of Technology, 19/27 Skłodowskiej–Curie Street, 90-570 Lodz, Poland; nina.tarzynska@lit.lukasiewicz.gov.pl (N.T.); anna.bednarowicz@lit.lukasiewicz.gov.pl (A.B.); 3 Laboratory of Biodegradation and Microbiological Research, ŁUKASIEWICZ Research Network—Lodz Institute of Technology, 5/15 Brzezińska Street, 92-103 Lodz, Poland; piotr.kaczmarek@lit.lukasiewicz.gov.pl

**Keywords:** biopolymers, surface modification, surface properties, structural analysis, biomaterials, titanium dioxide, micro-tomography

## Abstract

**Highlights:**

**What are the main findings?**

- ALD enables uniform TiO_2_ coatings on porous HA foams at nanometric scale.
- TiO_2_ layer protects HA from aqueous degradation and may reduce microbial risk.
- Silk decreases foam hydrophilicity; elastin enhances polarity and water uptake.
- Wettability and solubility depend on distinct mechanisms in HA and modified foams.

**What are the implications of the main findings?**

- ALD coatings improve scaffold stability for long-term biomedical use.
- TiO_2_ barrier may support antimicrobial properties, reducing infection risk.
- Protein modifiers allow tuning of foam wettability for tissue engineering.

**Abstract:**

The aim of this study was to evaluate the effect of surface modification of porous hyaluronic acid (HA)-based materials with a titanium dioxide (TiO_2_) layer deposited via atomic layer deposition (ALD) on the selected structural, physicochemical, and antimicrobial properties of materials intended for applications in regenerative medicine. The obtained HA-based materials, enriched with silk and elastin, were analyzed in terms of their rheological behavior, wettability, solubility, and resistance to colonization by clinically relevant bacterial pathogens (*Staphylococcus aureus*, *Klebsiella pneumoniae*) and environmental filamentous fungi (*Aspergillus niger*, *Chaetomium globosum*). The results demonstrated that even a thin, continuous TiO_2_ layer formed after 200 ALD cycles reduced the hydrophilicity of the foams, indicating improved durability in aqueous environments. Microbiological tests confirmed enhanced antimicrobial properties of the foams after TiO_2_ modification—showing inhibition of both tested bacterial strains and *C. globosum* within 24 h. These findings suggest that surface functionalization of hyaluronic acid-based foams with a TiO_2_ layer can improve both their environmental stability and, to some extent, reduce microbiological risk, while preserving the layered-porous structure of the foams, which is advantageous for biomedical applications.

## 1. Introduction

In recent years, the search for novel and functional biomaterials for applications in regenerative medicine has gained particular importance in the context of growing clinical needs, shortages of transplantable tissues, and increasing complications related to implant-associated infections, immune rejection, and wound dressing changes. Natural-origin polymers such as collagen, chitin, gelatin, alginate, and especially hyaluronic acid (HA) have attracted significant interest due to their excellent biocompatibility, biodegradability, and structural similarity to the extracellular matrix (ECM) of native tissues. These properties make natural polymer-based materials a promising platform for applications in tissue engineering and regenerative medicine and are the subject of numerous ongoing research efforts [1,2,3]. Collagen, as a key ECM component, provides mechanical strength and regulates cellular expression through its triple-helical structure [4,5,6]. Chitin derivatives, particularly chitosan, support tissue regeneration and exhibit antibacterial and anti-inflammatory properties [7,8,9]. Hyaluronic acid (HA) plays a central role in angiogenesis and cell proliferation, making it essential for soft tissue regeneration [10]. Silk and elastin are natural polymers with unique biological properties. Silk fibroin combines high mechanical strength, biocompatibility, and controlled biodegradability, making it highly promising for tissue engineering. Elastin, responsible for tissue elasticity, is crucial for regenerating structures such as blood vessels, lungs, and skin [11,12,13]. However, HA and its derivatives deserve particular attention as primary components of polymeric matrices, despite challenges linked to their hydrophilic nature. This glycosaminoglycan, a major ECM constituent, exhibits properties ideal for tissue engineering [14]. HA supports wound healing, angiogenesis, and immune modulation due to its ability to bind large amounts of water and form viscoelastic structures. These features create an optimal environment for cell migration, proliferation, and differentiation. As an endogenous molecule, HA is highly biocompatible and non-immunogenic, while its enzymatic biodegradability ensures controlled scaffold degradation aligned with tissue regeneration. HA also promotes macrophage polarization toward the M2 phenotype, accelerating healing and reducing inflammation [15,16,17,18].

Despite these advantages, natural polymers often lack mechanical strength, stability in physiological conditions, and resistance to microbial colonization [19]. HA faces similar limitations, including rapid dissolution, low mechanical stability, and excessive hydrophilicity, restricting its use in demanding environments such as chronic wounds or long-term scaffolds [20,21,22,23]. These challenges, however, drive innovation in HA modification and stabilization [24,25,26]. Its hydrophilic nature, often seen as a drawback, is beneficial for biomedical applications: HA hydrogels ensure nutrient diffusion, waste removal, and cell viability in 3D structures. Moreover, HA acts as a reservoir for growth factors and cytokines, supporting regenerative processes [27,28,29]. In response to these challenges, surface modification strategies are being intensively developed to enhance the functionality of these materials. Surface modification using titanium dioxide (TiO_2_) appears particularly promising due to the unique properties of this material [30]. One of the most promising approaches involves coating with a TiO_2_ layer using advanced techniques such as atomic layer deposition (ALD). TiO_2_ coatings are characterized by high biocompatibility, antibacterial properties, photocatalytic activity, and the ability to precisely control thickness and morphology. Literature reports indicate that the deposition of thin TiO_2_ layers on metallic biomaterials significantly improves resistance to bacterial colonization, while simultaneously enhancing mechanical strength and cellular affinity. The ALD technique, in particular, enables highly precise, nanometric control over the thickness and uniformity of TiO_2_ coatings, while preserving the integrity of porous internal structures [31,32,33]. Since this modification can be performed at various structural levels—from the incorporation of nanoparticles into the bulk material, through surface coating, to surface functionalization—each approach can differently influence the morphological structure, physicochemical properties, and biological response of composite materials. The latter, i.e., surface functionalization, allows for fine-tuning of interactions between the material and the biological environment by introducing specific functional groups on the surface. Of particular importance for medical applications are the antibacterial properties of TiO_2_-modified materials, which may contribute to reducing the risk of peri-implant infections—one of the major challenges in contemporary regenerative surgery [34,35]. The biocompatibility aspects of TiO_2_-modified materials are the subject of intensive research, especially in the context of the potential cytotoxicity of nanoparticles. However, studies on the impact of TiO_2_ modification on tissue regeneration processes show promising results in terms of stimulating cell proliferation, differentiation, and angiogenesis [36,37].

The structural characterization of biomedical materials—including parameters such as porosity, pore architecture, surface and internal topography, layering, and wall continuity—plays a fundamental role in shaping the biological functionality of materials intended for regenerative medicine. These properties determine how cells interact with the material, influencing adhesion, migration, proliferation, and differentiation. For instance, materials with high porosity and appropriately designed pore geometry promote angiogenesis and facilitate the transport of nutrients and gases, which is crucial for the regeneration of metabolically demanding tissues. Moreover, micro- and nanostructures present on the material surface can modulate cellular responses through mechanical and chemical cues, activating signaling pathways responsible for repair processes [38]. Structural integration of the material with the cellular scaffold can also influence the local immune response, minimizing the risk of inflammatory reactions and supporting the transition of macrophages toward the M2 phenotype, which favors regeneration [39,40]. High precision in designing the structure and surface of materials enables the creation of scaffolds with tailored morphologies suited to specific tissue types [41]. This approach not only provides mechanical support for regenerating structures but also allows the material to actively participate in biological processes, making it not merely a passive carrier but an active contributor to regeneration. Consequently, structural optimization of biomaterials represents one of the key directions in the development of modern tissue engineering, enabling the creation of intelligent therapeutic systems with high clinical efficacy [42,43].

**Figure 1:**
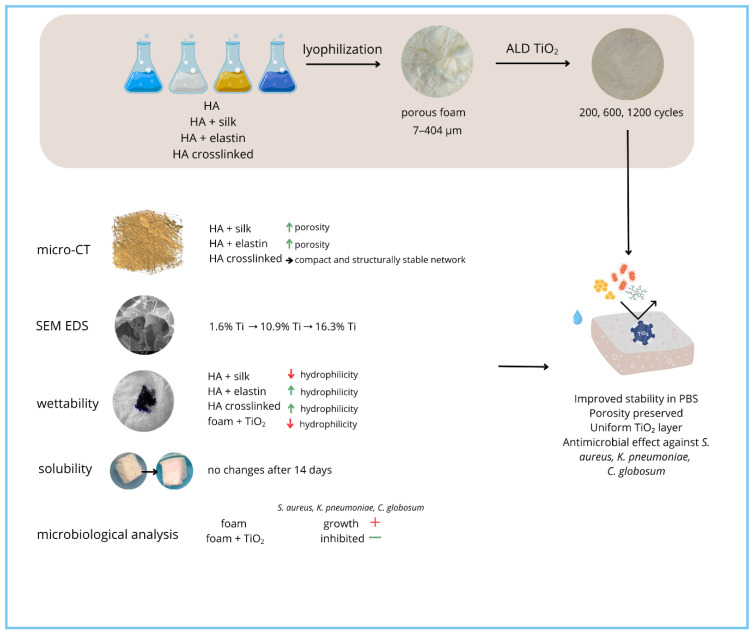
The overview of the experimental workflow for TiO_2_ deposition and foam characterization.

Based on insights gathered from the literature review, the aim of this study was to conduct a comprehensive analysis of the impact of titanium dioxide surface modification on the internal structure, porous architecture, and selected physicochemical and biological properties of layered and porous materials based on natural polymers, according to the experimental workflow shown in Figure 1.

Particular attention was paid to internal structure, pore size, and tissue thickness, as well as the internal morphology of the material. These findings were correlated with biological studies and the behavior of the materials under simulated physiological conditions. The study assessed, among other aspects, the quality and distribution of the titanium dioxide layer and the influence of the resulting parameters on solubility, wettability, and antimicrobial properties of non-metallic biomaterials based on hyaluronic acid. The presented research included a full analysis of morphological changes (micro-CT, SEM), stability under biologically simulated conditions, and antimicrobial efficacy against clinically challenging pathogens, representing a novel approach in the context of current literature.

## 2. Materials

Cosmetic-grade hyaluronic acid (HA, sodium hyaluronate) was purchased from Contipro Biotech (Pardubický, Czechia) in the form of a sodium salt with a molecular weight of M = 2.0–2.2 MDa, as well as in a crosslinked form with a monomer molecular weight of M = 300 kDa. The molecular weight of the non-crosslinked polymer was selected based on preliminary studies and previous work by the research team [44,45,46,47]. The method of hyaluronic acid crosslinking has been patented by Contipro Biotech and, in general terms, involves oxidizing the hyaluronic acid to a polyaldehyde, which is then crosslinked at a 1:5 ratio. The final product is a water-soluble powder that requires no preservatives or stabilizers. In this study, silk and elastin, both of cosmetic-grade purity, were used as active additives potentially influencing the structure and properties of the foams. These proteins were purchased from Proteina Wytwórnia Naturalnych Białek (Łódź, Poland).

## 3. Methods

### 3.1. Sample Preparation

#### 3.1.1. Preparation of Polymeric Solutions

All polymers used in the study were supplied in powder form, soluble in distilled water at room temperature. After weighing the appropriate amount of polymer, the material was transferred into a beaker and subjected to mechanical stirring (RPM = 150, temperature = 20 °C) for 6–8 h. If the solutions showed signs of air entrapment, they were left to degas overnight before being used for testing or poured into molds for freezing. Based on previous analyses, the following model was selected for the study: base polymer concentration (molecular weight 2.0–2.2 MDa) 2%, in both non-crosslinked and crosslinked variants; active additive concentration: 1% or 2%, for silk and elastin, respectively, and reference samples consisted of pure (non-enriched) base polymer solutions. The following systems were selected:

1. HA 2%, 2.0–2.2 MDa
2. HA 2%, 2.0–2.2 MDa + 1% silk
3. HA 2%, 2.0–2.2 MDa + 2% elastin
4. HA 2%, crosslinked

#### 3.1.2. Rheological Properties

Rheological characteristics are crucial for the further processing of polymeric solutions. Depending on the processing method used (e.g., electrospinning or freeze-drying), rheological parameters can influence the morphological properties of the resulting structures (e.g., layer thickness, porosity, etc.). Rheological measurements were performed using a rotational rheometer (Anton Paar, model RheolabQC, Luton, Great Britain). Data were recorded using Rheoplus/322 V3.21 software. Measurements were conducted over a shear rate range of 0.2 to 160 s^−1^ using a cylinder type H. The temperature was maintained at 25 °C using a thermostatic bath. To determine the basic rheological parameters, the Ostwald–de Waele power-law model [48] was applied to approximate the behavior of the polymer fluid. The viscosity of the fluid was defined according to Newton’s law, which describes the relationship between the tangential force applied to a surface, the surface area, and the resulting velocity gradient perpendicular to the surface, which is described with Equation (1):

(1) $$F=ŋ\cdot A\frac{dw}{dy}\;\;\;\tau =ŋ\frac{dw}{dy}$$

where

*F*—the force tangential to the surface, N,

η—dynamic viscosity coefficient, viscosity Pa·s,

*A*—surface area, to which the applied force has been, m^2^,

$\frac{dw}{dy}$—a fluid velocity gradient in a direction perpendicular to the surface of 1·s^−1^,

τ—shear stress, Pa.

One of the simplest models describing the flow of rheologically stable liquids showing no yield point is the power model of Ostwald de Waele, which is described by Equation (2):

(2) $$\tau =k\cdot {\left(\frac{dw}{dy}\right)}^{n}=k\cdot \;{\left(\frac{dy}{dt}\right)}^{n}$$

where

*τ*—shear stress, Pa,

$\dot{\gamma }=\frac{dy}{dt}=\frac{dw}{dy}$—shear strain rate, s^−1^,

*k*—an indicator of consistency, apparent viscosity, Pa·s^n^,

*n*—a characteristic indicator of flow rate—flow behavior index.

For Newtonian fluids (all gases and liquids, most of low molecular weight liquids) *n* = 1 and then, K is the apparent viscosity.

#### 3.1.3. Lyophilization

To obtain porous structures, commonly referred to as sponge-like structures, the polymer solutions were frozen at −45 °C and subsequently lyophilized using a freeze dryer (Labconco, Kansas City, MO, USA) under conditions selected based on preliminary studies [44,45,46,47]: chamber temperature −50 °C, shelf temperature −50 °C, duration 12–16 h, and sublimation pressure 0.26 mbar.

#### 3.1.4. Surface Modification—TiO_2_ Deposition

Surface modification was performed to alter the surface properties of the foams, reduce their solubility in water and biological media, and potentially limit microbial colonization—aiming to achieve a minimal antimicrobial effect. The deposition process was carried out on porous substrates using a plasma-enhanced atomic layer deposition (PE-ALD) reactor (PicoSun, model R-200 Advanced, Uusimaa, Finland). The following precursors were used: titanium tetrachloride (TiCl_4_) with purity above 99.5% supplied by EpiValence (Redcar, Cleveland, UK), and deionized water prepared using a Thermo Scientific water purification system (Thermo Fisher Scientific, Waltham, MA, USA). The deposition parameters were as follows: substrate temperature: 60 °C, precursor temperature: 20 °C, inert gas flow (nitrogen 5.0) through the reaction chamber: 150 sccm, inert gas flow on the TiCl_4_ line: 120 sccm, inert gas flow on the H_2_O line: 150 sccm (standard cubic centimeters per minute), TiCl_4_ pulse time: 0.1 s, TiCl_4_ purge time: 15 s, H_2_O pulse time: 0.1 s and H_2_O purge time: 20 s. Deposition was performed on the provided material in 200, 600, and 1200 cycles. The deposition process was evaluated by analyzing the thickness of the TiO_2_ layer on a reference material (silicon wafer) using a FR-Basic reflectometer (ThetaMetrisis, Athens, Greece). The thickness of the TiO_2_ layer was determined using by analyzing the reflectance spectrum of the reference silicon wafer in the wavelength range of 400–800 nm. The instrument compares the measured reflectance curve with theoretical models to calculate film thickness accurately [49].

### 3.2. Sample Analysis

#### 3.2.1. FTIR Absorption Spectroscopy of Deposited Titanium Dioxide Layers

The comparative analysis using infrared absorption spectroscopy aimed to identify potential differences in the quality (thickness) of TiO_2_ layers deposited via ALD on silicon wafers. Samples were analyzed both without TiO_2_ deposition and with deposition performed in 200, 600, and 1200 cycles. Differences in the molecular structure of the surface layer were assessed by identifying new absorption peaks originating from the deposited TiO_2_ [50].

The FTIR-ATR reflection method was applied using a NICOLET 6700 spectrometer (Thermo Fisher Scientific, Waltham, MA, USA). equipped with a Smart iTR accessory and a diamond crystal with a 45° angle of incidence. Spectra were recorded in the wavenumber range of 4000–600 cm^−1^ with a resolution of 4 cm^−1^, in the format A = f(1/λ). A total of 32 spectra were collected within the designated range. For the analysis of spectrograms and identification of characteristic bands associated with TiO_2_ (in the range of 600–1500 cm^−1^), the OMNIC 0.8 software (Thermo Fisher Scientific, Waltham, MA, USA) was used.

#### 3.2.2. Microscopic Analysis

Morphological structure and elemental surface composition of the materials were evaluated using scanning electron microscopy (SEM) and energy-dispersive X-ray spectroscopy (EDS). The analysis was conducted using a Nova NanoSEM 230 scanning electron microscope (FEI, Hillsboro, OR, USA) equipped with a field emission gun (FEG) and an Apollo 40 SDD EDS detector (EDAX). The following operating parameters were used: vacuum mode: LowVac 70 Pa, accelerating voltage (HV): 10 kV, detector: LVD—secondary electrons. The EDS measurements were performed with pressure correction using the ViPQuant software (VIP Software, Lakeland, FL, USA), and quantitative analysis was based on the ZAF correction procedure. The studies were conducted under low vacuum conditions, with beam energy ranging from 10 to 15 keV, detector voltage of 70 kV, and spot size of 3 kV.

#### 3.2.3. X-Ray Microtomography

The aim of the study was to characterize selected elements of the internal structure of the foams using X-ray microcomputed tomography. Parameters such as porosity, wall thickness, and pore size were determined, and 3D models of the porous polymer structures were generated. The analyses were performed using a SkyScan 1272 micro-CT scanner (Bruker, Kontich, Belgium). All samples were scanned under identical conditions defined by the following parameters: source voltage: 50 kV, source current: 200 μA, resolution: 4032 × 2688 pixels, pixel size: 6.6 μm, exposure time: 950 ms, rotation step: 0.2°, frame averaging: 5 images per angle, filter: none.

#### 3.2.4. Wettability

Wettability assessment was performed using a modified Tegewa Drop Test [51]. A 3 mL aqueous solution of methylene blue (0.2% *w*/*v*) was applied to the foam surface. Photographs were taken immediately after contact and after 30 s for different foam variants and the stain area was measured using ImageJ software (version 1.54k of September 2024). The analysis began by converting the images to grayscale and adjusting contrast to clearly define the stain boundaries. A thresholding tool was then applied to isolate the stained region. Using the “Analyze → Measure” function, ImageJ calculated the stain area in pixels. This quantitative approach allowed for an objective comparison of wettability between different foam variants [52].

#### 3.2.5. Solubility Testing

Samples after TiO_2_ deposition and a reference sample were subjected to solubility testing in PBS solution at room temperature. Each sample was placed in a Petri dish and covered with approximately 10 mL of PBS. Evaluation was based on visual observation of changes in the appearance of the samples exposed to PBS, particularly changes in the external structure, edge integrity, and any other alterations occurring during the test period.

#### 3.2.6. Antibacterial Testing

Antibacterial properties were assessed using qualitative method according to PN-EN ISO 20645:2006 (against *Staphylococcus aureus* and *Klebsiella pneumoniae*)—Diffusion method for determining antibacterial activity [53]. Antifungal properties were evaluated according to PN-EN 14119:2005, section 10.5 (B2)—Antifungal activity, diffusion test, visual method [54]. Biological tests were conducted at the accredited Laboratory of Biodegradation and Microbiological Testing, part of the ŁUKASIEWICZ Research Network—Łódź Institute of Technology, Poland (accreditation certificate no. AB 388). The laboratory operates under a quality management system compliant with PN-EN ISO/IEC 17025:2018-02. The selected panel includes pathogens critical for validating the safety and efficacy of novel regenerative biomaterials: *K. pneumoniae* and *S. aureus* represent clinically relevant bacterial strains, while *A. niger* and *C. globosum* are major fungal threats to biomaterial stability, biocompatibility, and sterility in both clinical and laboratory environments [55,56]. This approach aligns with current standards and expectations for wound care products and tissue engineering scaffolds intended for direct contact with biological systems. A brief overview of the test panel is presented in Table 1.

## 4. Results

The study investigating the impact of titanium dioxide surface modification was based on the selection of polymer solutions with 2% concentration of the base polymer (hyaluronic acid, HA), enriched with 1% silk and 2% elastin, as well as a non-enriched solution of crosslinked HA used for comparative purposes. The selection was guided by rheological properties and visual–organoleptic analysis of the lyophilized foams. Table 2 presents a description and photographic documentation of the samples, along with their rheological characteristics, including apparent dynamic viscosity and the flow behavior index (*n*).

Rheological analysis revealed that sodium hyaluronate solutions with a molecular weight of 2.0–2.2 MDa exhibit non-Newtonian shear-thinning behavior (*n* < 1). The addition of 1% silk or 2% elastin resulted in a reduction in their apparent dynamic viscosity. Following this, the surface modification process using titanium dioxide was carried out. The deposition process was evaluated by analyzing the thickness of the TiO_2_ layer on a reference material. The thicknesses of the TiO_2_ layer obtained after a defined number of deposition cycles (*n*) are presented in Table 3.

**Figure 2:**
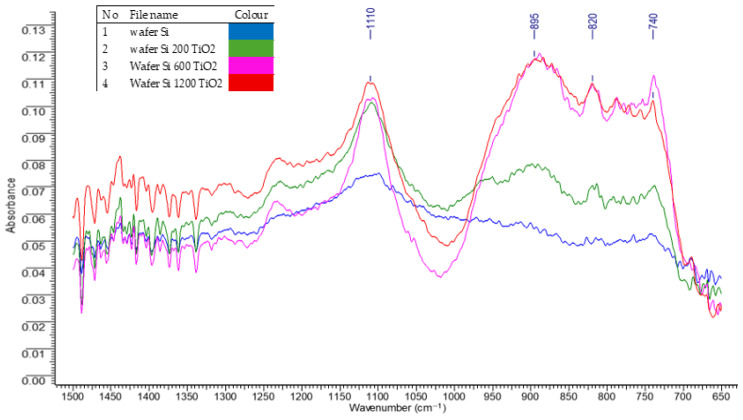
The spectrograms characterizing the chemical structure of the tested samples—silicon wafers with a different number of cycles applied.

The obtained measurement results are consistent with literature data concerning TiO_2_ deposition via the atomic layer deposition (ALD) method [57,58]. The thickness of the TiO_2_ layers was found to be proportional to the number of deposition cycles. For all samples, the thickness per cycle was approximately 0.26–0.29 Å at a process temperature of 60 °C. Comparative studies and analysis using infrared radiation absorption spectroscopy were conducted to determine potential differences in the quality (thickness) of TiO_2_ layers deposited via ALD on silicon wafers using varying numbers of cycles (*n*). The spectrograms illustrating the chemical structure of the tested silicon wafers with deposited TiO_2_ layers are presented in Figure 2.

The spectrograms revealed absorption bands characteristic of TiO_2_ in the range of 700–1100 cm^−1^, along with the emergence of new signals at 1110, 895, 820, and 740 cm^−1^, which intensified with an increasing number of deposition cycles which correspond to the previous works [59,60]. This indicates effective formation and progressive thickening of the titanium dioxide layers. The intensification of absorption bands up to approximately 600 deposition cycles suggests that the TiO_2_ layer reaches sufficient coherence and surface coverage, which is essential for ensuring a uniform and functional coating. Beyond 600 cycles, further increases in the number of cycles result in layer duplication, indicating continued growth in thickness rather than a significant improvement in coating quality. This may lead to changes in the physical and chemical properties of the layer.

The morphological structure and elemental chemical composition of the foams after TiO_2_ deposition were evaluated using scanning electron microscopy (SEM) and energy-dispersive X-ray spectroscopy (EDS), as presented in Table 4. The aim of the study was to assess the impact of the number of ALD cycles on the surface morphology and chemical composition of the tested materials. For the analysis, sample foam no. 1 was selected and subjected to deposition in three cycle variants.

The energy-dispersive X-ray spectroscopy (EDS) was performed to determine the quantitative composition of the coatings in terms of weight percentage. The results of the analysis are presented in Table 5.

The SEM-EDS analysis conducted on foams modified with TiO_2_ via the ALD method clearly demonstrated the influence of the number of deposition cycles on both the surface morphology and chemical composition of the analyzed materials. Microscopic observations (SEM) revealed progressive changes in the foam surface with increasing ALD cycles. After 200 cycles, the surface exhibited the presence of an initial, irregular TiO_2_ layer, whereas at 600 and 1200 cycles, the oxide coating became increasingly uniform and finely distributed, indicating a gradual increase in thickness and continuity of the deposited titanium dioxide layer. EDS analysis confirmed effective and progressive incorporation of titanium into the foam surface. The titanium content increased significantly with the number of cycles: from 1.64% (200 cycles) to 10.89% (600 cycles) and 16.30% (1200 cycles). Simultaneously, the proportion of organic matrix elements—carbon and oxygen—decreased systematically (carbon: from 54.38% to 43.00%; oxygen: from 41.01% to 36.02%), which correlates with the gradual coverage of the polymer structures by the inorganic TiO_2_ layer. The minor presence of sodium, sulfur, and chlorine suggests possible trace residues from previous synthesis steps or contamination. Elemental distribution maps clearly showed an increase and homogenization of the titanium signal with the number of ALD cycles, confirming the effectiveness of this method in uniformly enriching the foam surface with a titanium dioxide layer.

**Figure 3:**
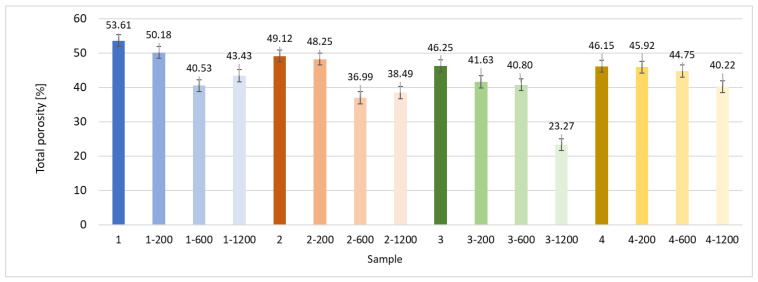
Total porosity of the selected samples: samples from 1 to 4 with four variants of deposition (*n* = 0, *n* = 200, *n* = 600 and *n* = 1200).

**Figure 4:**
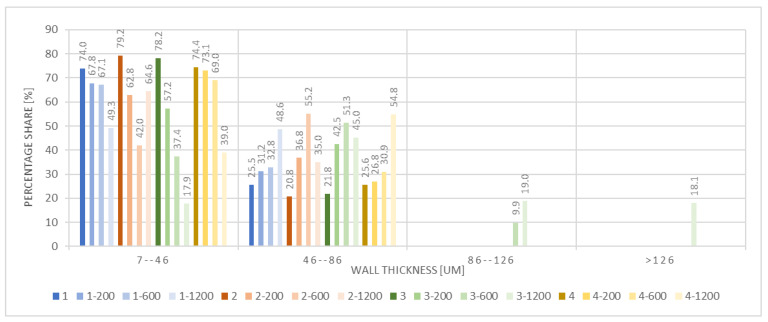
Wall thickness distribution of the selected samples.

**Figure 5:**
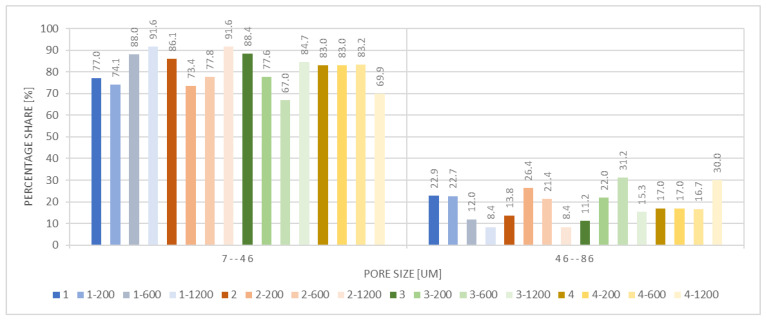
Pore size distribution of the selected samples.

The next stage involved the analysis of internal structure and porous architecture using X-ray microcomputed tomography. The aim of the study was to characterize selected structural parameters of the foams based on 3D and 2D images obtained through high-resolution scanning. The results of the analysis are presented in Figure 3, Figure 4 and Figure 5 and Table 6.

Figure 3 illustrates the total porosity of the samples. The highest porosity was observed for the base sample 1 (53.61%), while the lowest was recorded for sample 3-1200 (23.27%). Samples 2, 2-200, 2-600, and 2-1200 exhibited moderate porosity levels ranging from 36.99% to 49.12%. In contrast, the modified samples showed greater variability—porosity in series 3 and 4 ranged between 23.27% and 46.25%, suggesting a significant influence of process variables on morphology and potentially on the transport properties of the materials. It can therefore be concluded that the porosity of the foams spans a wide range, from approximately 23.3% to 53.6%, indicating a diverse porous structure. Notably, nearly 100% of the porosity is open porosity, which suggests a well-connected pore system conducive to the flow of gases or liquids within the material. In the context of TiO_2_ modification, the preservation of open porosity is crucial for applications involving bioactive materials.

Figure 4 presents the percentage distribution of wall thickness in the analyzed foams. The highest proportion was observed for walls with a thickness ranging from 7 to 46 µm, accounting for 57% to 74% of the total structure. Thicker walls, ranging from 46 to 86 µm, constituted between 17.9% and 48.6%, depending on the sample. Wall thicknesses exceeding 86 µm were rare and occurred almost exclusively in sample 3-1200, reaching up to 18.1%. The variation in wall thickness among the samples may indicate the influence of the type and concentration of the modifier (silk or elastin) on the development of the wall network and the resulting porosity.

Figure 5 presents the percentage distribution of pores of different sizes (7–46 µm and 46–86 µm) across all analyzed samples. The vast majority of pores in all samples fell within the 7–46 µm range, with volumetric contributions ranging from 67% to 91.6%, indicating a clear dominance of small pores in the material structure. The proportion of pores within the 46–86 µm range was significantly lower, ranging from 8.4% to 31.2%. The highest percentage of larger pores was observed in samples 3, 3-1200, and 4-200, suggesting a locally more open structure.

Titanium dioxide modification did not show any clear depletion or thickening of the walls based on the available data, which suggests that the ALD process preserves the integrity of the foam’s fundamental structural morphology. The pore distribution corresponds to the typical structure of polymeric foams with controlled porosity, ensuring optimal functional properties. TiO_2_ deposition does not negatively affect pore size, allowing the material’s transport properties to be maintained, as shown elsewhere [61]. However, modification of the foam composition results in certain differences in pore distribution and wall thickness, which directly influence overall porosity and the potential functional properties of the material. Samples with a higher proportion of small pores and thinner walls exhibited greater total porosity, which may be advantageous in applications requiring high permeability or large specific surface area. Structures with more heterogeneous wall thickness may potentially demonstrate greater mechanical resistance but lower overall porosity. Table 6 presents 3D X-ray visualizations of the foams showing internal structure, along with 2D visualizations of foam edges.

The microtomographic analysis presented in Table 6 reveals a diverse internal morphology, while 2D edge visualizations allow for the assessment of wall continuity and pore distribution in cross−section. Sample 1-600 (blue) exhibits a structure with relatively high porosity, confirmed by numerous small pores visible in both 3D images and 2D cross-sections. The observed walls are thin and evenly distributed, indicating a well-developed pore network with dominant pore sizes in the range of 7–46 µm. Such a structure promotes high permeability and may be advantageous in applications requiring a large specific surface area. Sample 2-200 (purple) and sample 3-600 (red) are characterized by a more heterogeneous structure, with the presence of both thin and thicker walls. The 2D visualizations reveal localized material densification, which may contribute to a reduction in total porosity. The presence of larger pores (46–86 µm) is more pronounced than in sample 1-600, suggesting a locally more open structure but also lower material uniformity. However, sample 3 shows the highest degree of layer packing and structural homogeneity. In contrast, sample 4-600 (yellow) displays an intermediate structure—3D images show moderate porosity with a uniform pore distribution, while 2D visualizations indicate the presence of both thin and thick walls with noticeably greater spacing between them. The pore distribution is relatively homogeneous, which may reflect a well-controlled modification process. In summary, the foam structures differ significantly in terms of porosity, wall thickness, and pore distribution, confirming the influence of various processing parameters. Samples with a higher proportion of small pores and thinner walls (e.g., 1-600) exhibit greater total porosity, which may be beneficial in applications requiring high permeability.

**Figure 6:**
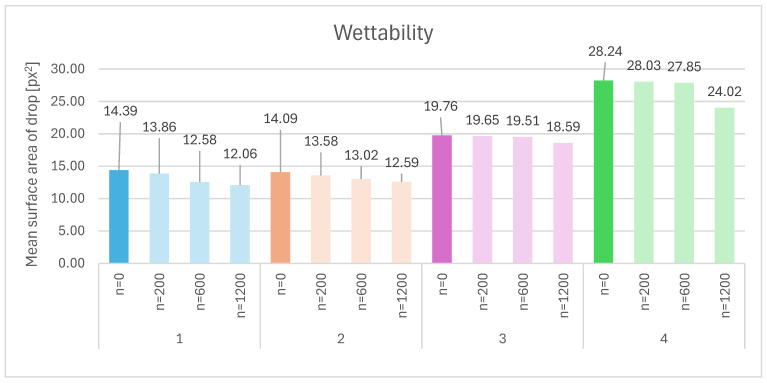
The wettability of samples after 30 s of observation based on the modified Tegewa test.

The surface property analysis was based on solubility testing of the foams in a medium simulating biological conditions, in this case using PBS solution, and wettability testing using a modified Tegewa Drop Test method [51]. The results of the wettability tests are presented in Figure 6, and a comparative analysis of wettability measurements is shown in Table 7.

The wettability results presented in Figure 6 and Table 7 show that sample 4 (crosslinked HA) exhibits the highest hydrophilicity, as confirmed by the largest droplet surface areas (28.24 at *n* = 0 and 24.02 at *n* = 1200). This indicates that, despite its compact structure and lower porosity, the surface of this foam is highly hydrophilic. This effect may be attributed to the presence of polar groups formed during the crosslinking process, which enhance water affinity. Sample 3 (HA + 2% elastin) also demonstrates elevated hydrophilicity (19.76–18.59 px^2^), significantly higher than samples 1 and 2. Elastin, as a hydrophilic protein, may increase surface wettability through the presence of amino and carboxyl groups that promote interaction with water. Despite its more heterogeneous structure and locally larger pores, this sample maintains good wettability, which may be beneficial for biological applications. Sample 2 (HA + 1% silk) and sample 1 (pure HA) exhibit the lowest hydrophilicity (14.09–12.59 and 14.39–12.06, respectively). In the case of pure HA, the lower wettability may result from a limited number of polar groups available on the surface and a more open but less reactive structure. The addition of silk at a low concentration does not significantly improve wettability, suggesting that the surface modification effect may be concentration-dependent.

When comparing the structural data from Table 6 with the wettability results, it becomes evident that hydrophilicity is not directly correlated with porosity. For example, sample 1-600 (pure HA) has high porosity but low wettability, whereas sample 4-600 (crosslinked HA) has low porosity but the highest wettability. This indicates that chemical composition and surface modifications have a greater impact on wettability than the porous structure alone. Samples 2 and 3 differ from samples 1 and 4 not only in structure but primarily in composition. The presence of proteins (silk, elastin) affects surface properties, with elastin (sample 3) showing a stronger hydrophilic effect than silk (sample 2), likely due to its more polar nature and greater capacity for water interaction. Subsequently, the solubility of the foams in a medium simulating biological conditions was analyzed, with the results presented in Table 8.

Based on the observations, it can be concluded that the TiO_2_ deposition process significantly reduced the dissolution and degradation of the material. After a 16-day period, the unmodified sample resembled a hydrogel with no visible structure, whereas the deposited samples, despite considerable swelling, retained their form. The solubility analysis of hyaluronic acid-based foams in PBS solution clearly demonstrates the influence of the number of TiO_2_ deposition cycles on the material’s stability in aqueous environments. Unmodified foams (*n* = 0 cycles) exhibited a high degree of solubility in PBS. As early as three days into incubation, a reduction in volume and visible signs of edge dissolution, along with increased transparency, were observed. This phenomenon intensified over time—after nine days, the changes progressed, and by day fourteen, the foam had lost most of its volume and structural integrity, indicating low durability in aqueous conditions. Foams with a TiO_2_ layer (*n* = 200, 600, 1200 cycles) showed significantly enhanced resistance to aqueous solutions. In all modified variants, no substantial morphological changes were observed throughout the incubation period—the structure and size of the foams remained unchanged even after 14 days of contact with PBS. Even a small number of cycles (*n* = 200) effectively protected the material from dissolution, and further increases in cycle number did not additionally improve solubility resistance within the observed timeframe. These findings suggest that even a thin, uniform TiO_2_ layer obtained via ALD can contribute to limiting water phase penetration into the HA foam structure, thereby blocking hydrolysis and surface erosion processes, and consequently reducing internal degradation. The results indicate potentially high effectiveness even at low cycle numbers, which is a significant advantage for future applications of biomaterials requiring long-term stability in biological environments. The results of the antimicrobial tests are presented in Table 9.

The results of microbiological testing, presented in Table 9, reveal significant changes in the biological activity of the material within 24 h of application, depending on the degree of modification (*n* = 0 vs. *n* = 1200). In its unmodified state (*n* = 0), the foam exhibited no antimicrobial properties—intense growth of all tested microorganisms was observed, including Gram-negative bacteria (*Klebsiella pneumoniae*), Gram-positive bacteria (*Staphylococcus aureus*), filamentous fungi (*Aspergillus niger*), and saprophytic mold (*Chaetomium globosum*). In each case, no inhibition zones or reduction in colonization intensity were observed compared to the control, indicating a lack of antimicrobial activity on the surface of the base foam. Similar results were obtained for the sample with 600 deposition cycles (*n* = 600). However, after intensive modification (*n* = 1200), sample 1 demonstrated antimicrobial activity. For both bacterial strains (Gram-negative and Gram-positive), complete inhibition of growth was observed, along with the presence of inhibition zones exceeding 1 mm, indicating strong antibacterial effects within 24 h. In the case of *Aspergillus niger*, growth was limited to only 25% of the surface, suggesting that the modification had no significant impact on this microorganism. For *Chaetomium globosum*, no growth was observed under microscopic examination at 50× magnification, confirming a certain degree of antifungal activity. In the context of previous structural and surface results, it can be concluded that modification of foam 1 (*n* = 1200) leads not only to reduced wettability but also to enhanced surface bioactivity.

## 5. Discussion

In this study, an analysis was conducted of the structural properties—including surface and internal characteristics—physicochemical parameters, and microbiological behavior of non-metallic materials, specifically foams based on hyaluronic acid (HA), modified with a titanium dioxide (TiO_2_) layer using the atomic layer deposition (ALD) method. The reference material selected for the study was pure, non-crosslinked HA at a concentration of 2%, which was compared with samples containing unmodified HA and samples enriched with modifiers: silk (sample 2) and elastin (sample 3). In the context of polymer solution characterization and its influence on the resulting structure and properties, it should be noted that solutions exhibiting non-Newtonian shear-thinning behavior (*n* < 1), with apparent viscosities ranging from 42.259 to 55.959 Pa·sⁿ, allow for a partial understanding of the interactions between solution properties and final foam morphology. For example, the reduction in apparent viscosity following the addition of 1% silk or 2% elastin may be associated with protein–polymer interactions that influence pore architecture during the lyophilization process. A fundamentally different molecular organization and structural profile of the resulting foam was indicated, as confirmed by 3D tomographic visualizations and 2D edge layer imaging. These findings support the notion that differences in rheological properties affect pore formation mechanisms during lyophilization, which in turn may influence the uniformity of the deposited titanium coating. The addition of modifiers (1% silk, 2% elastin) resulted in a reduction in the consistency index (k) compared to the base HA 2% solution, indicating lower initial viscosity. Simultaneously, the flow behavior index (*n*), associated with pseudoplasticity, increased—particularly after the addition of elastin (*n* = 0.45322)—suggesting greater viscosity instability under shear. Therefore, solutions with lower k and higher *n* (elastin, silk) enable the formation of structures with higher porosity and a greater proportion of fine pores, as confirmed by microtomography results: modified samples exhibited a higher number of pores in the 7–46 µm range and increased total porosity. The structure of foams obtained from different HA solutions is directly determined by the rheological properties of the liquid phase and molecular interactions between various chemical groups present in the polymer backbone and the added modifiers.

It should also be noted that the modification process using ALD did not compromise the internal structure of the foams, thus preserving their structural integrity and associated properties. Even after 1200 deposition cycles, no reduction in porosity or significant thickening of the walls was observed, while surface and biological properties were simultaneously improved. Microtomographic analysis showed no clear trend regarding the influence of the ALD process on porosity values, which ranged from 23.3% to 53.6%, with nearly 100% open porosity in all treated samples. The preservation of interconnected or open pore networks is particularly important in biomedical applications, where nutrient transport depends on maintaining porosity and pore connectivity. The observed pore size distribution (7–404 μm), with a predominance of pores in the 7–86 μm range, aligns with optimal dimensions for tissue engineering applications. Pore sizes between 100 and 300 μm facilitate cell ingrowth and are favorable for proper cell growth and proliferation, as well as for efficient nutrient transport and waste removal, making this range ideal for scaffolds used in tissue regeneration [62]. The variability in wall thickness across all samples (7–404 μm) indicates that the ALD process does not cause noticeable or measurable structural changes, nor significant alterations in layer or pore architecture. This contrasts with other surface modification techniques, such as plasma treatment or chemical vapor deposition, which often result in uneven coatings and may disrupt the microstructure of the material [63].

From the perspective of technological reproducibility and method validation, the uniformity of titanium dioxide (TiO_2_) deposits on polymer foam surfaces is fundamentally important for their functional properties and potential applications. This may also represent an advantage of the ALD method over other surface modification techniques, particularly in the context of non-metallic materials. Control over the homogeneity of TiO_2_ deposits can determine the effectiveness of surface modification, influencing both the durability and functionality of the foams in various technological and biomedical applications. Compared to other surface modification techniques, ALD stands out for its precise control over layer thickness, excellent coating uniformity—even on highly porous and rough structures—and the ability to deposit layers with nanometric thickness [64].

The wettability test results, performed using the modified Tegewa drop test, revealed a distinct influence of silk and elastin additives, as well as the degree of crosslinking, on the hydrophilic properties of hyaluronic acid (HA)-based foams. The presence of silk and elastin, along with the crosslinking of HA, significantly affected wettability. Silk fibroin consists of both hydrophilic blocks and large hydrophobic domains, particularly β-sheet structures. Numerous studies have shown that the incorporation of silk into HA hydrogels leads to the formation of ordered, hydrophobic aggregates that are poorly wettable due to a reduced number of polar groups available on the surface [65]. Silk fibroin contains both hydrophilic and hydrophobic domains, and at low concentrations (1%), the hydrophobic β-sheet regions may dominate surface interactions, thereby reducing overall wettability. Additionally, silk may interfere with HA chain hydration, further decreasing surface polarity. High and uncontrolled crystallization of β-sheets within the silk fibroin structure can limit adhesive and wetting properties, especially in pure or low-concentration systems. This phenomenon affects the morphology of the hydrogel network and its ability to interact with water [66]. In contrast, elastin, although it contains hydrophobic segments, has a large number of irregular fragments and a tendency to form more open, absorbent structures. Its integration with HA increases the number of polar groups exposed on the foam surface and promotes the formation of networks with greater free volume, facilitating capillary spreading and liquid absorption. This explains the larger stain areas observed on elastin-containing foams. Elastin (sample 3) introduces more polar amino acid residues (e.g., lysine, glutamic acid), enhancing surface hydrophilicity and water interaction. Crosslinked HA (sample 4) forms a dense network with exposed aldehyde and hydroxyl groups, increasing surface polarity [67]. Comparative studies show that elastin improves wettability and facilitates water transport and adhesion to the surface [68]. Contrary to intuition, crosslinking of HA in such foams may lead to networks with a greater number of accessible water-binding sites and exposed carboxyl groups, particularly when there is no strong transition to a gel phase and the open pore architecture is preserved. Crosslinking may also reduce coalescence or shrinkage during lyophilization, resulting in a firmer, absorbent foam rather than a compact hydrophilic mass. In many crosslinked HA systems, crosslinks stabilize the open structure rather than closing the network, so even with a reduced number of mobile chains, water mobility and absorption may increase or remain high. However, much depends on the chemical conditions and network architecture [69].

This non-obvious relationship between surface wettability and the solubility of hyaluronic acid (HA)-based foams requires reference to the current scientific understanding. The phenomenon observed in our study—where the foam made from pure HA (Sample 1) exhibits low wettability (i.e., a smaller droplet area compared to other samples, indicating lower hydrophilicity) while simultaneously being highly soluble in PBS—requires clarification. Although both wettability and solubility are related to water interactions, they are governed by different physical principles. Wettability reflects the surface energy and chemical functionality of the outermost layer of the material, whereas solubility is influenced by the presence of polar groups, surface roughness, and molecular orientation at the phase boundary. Solubility is a bulk property, determined by the thermodynamic compatibility of the polymer matrix with the solvent, the degree of crosslinking, molecular weight, and the entanglement of polymer chains [70]. A material may therefore be poorly wettable (low surface energy) but highly soluble if its polymer chains are hydrophilic and loosely entangled. Pure HA is a highly hydrophilic polyelectrolyte capable of absorbing large amounts of water due to its carboxyl and hydroxyl groups. However, in lyophilized foam, the surface may exhibit a low density of polar groups due to surface reorientation during sublimation drying, where hydrophilic groups migrate inward or form a “skin layer” with reduced hydration capacity. This results in low wettability in the Tegewa test. Upon immersion in PBS; however, the pure HA matrix rapidly absorbs water, leading to swelling and dissolution due to the absence of crosslinking and weak intermolecular interactions [71]. The TiO_2_ layer deposited via ALD acts as a “diffusion barrier,” limiting water penetration and preventing hydrolysis of HA chains. Even at a low number of cycles (*n* = 200), this coating stabilizes the foam structure, preventing water ingress and dissolution. In summary, the discrepancy between low surface wettability and high solubility in pure HA foam arises from the difference between surface and bulk interactions. While the surface may appear hydrophobic due to drying or surface modification, the bulk polymer remains highly hydrophilic and “unprotected,” leading to rapid dissolution when fully exposed to aqueous media.

While this study demonstrates successful TiO_2_ deposition and characterization, several aspects warrant further investigation. The long-term stability of the TiO_2_ coating under physiological conditions requires evaluation through extended in vitro and in vivo studies. Future research should aim to correlate the observed structural and compositional modifications with functional performance metrics, including mechanical properties, biocompatibility, and specific antimicrobial activity. Moreover, the scalability of the ALD process for larger scaffold geometries and different polymer compositions must be systematically assessed to establish the broader applicability of this surface modification approach. The conducted research contributes value to the current state of knowledge in the field of polymeric biomaterial surface modification. Unlike previous studies, this work integrates rheological analysis of HA solutions with an evaluation of the influence of protein-based modifiers (silk and elastin) and crosslinking processes on foam microstructure, porous architecture, wettability, and solubility under physiological conditions. Additionally, the application of the ALD method for TiO_2_ layer deposition on highly porous structures enabled the preservation of the material’s morphological integrity while imparting new functional properties, such as resistance to degradation and potential antimicrobial bioactivity. The integrated approach to these properties and their determining factors allowed for a deeper understanding of the mechanisms governing material behavior in biological environments and provides a foundation for further research and the potential design of functional tissue scaffolds.

## 6. Conclusions

This study demonstrates that the structural integrity and functional performance of HA-based foams are significantly enhanced by the application of Atomic Layer Deposition of TiO_2_. Unlike conventional surface treatments, ALD enables the formation of uniform, nanometric coatings that conform to the complex porous architecture without negatively affecting it. The TiO_2_ coating acts as an effective diffusion barrier, reducing degradation in physiological environments and mitigating the rapid solubility observed in uncoated HA foams. By stabilizing the surface, ALD prolongs structural integrity and offers potential for antimicrobial functionality and enhanced bioactivity, which is important for advanced tissue engineering scaffolds.

Although modifiers such as silk and elastin influence surface wettability, their role is secondary compared to the effect of TiO_2_ deposition. The coating compensates for limitations in hydrophilicity and structural resilience, causing that even foams with high wettability, thanks to the surface modification, can maintain functional performance to a certain point, despite the difficult biological conditions. These findings can suggest that ALD could be considered as a strategy for tailoring porous biomaterials, providing control at the nanoscale and solutions for next-generation biomedical applications.

Future research should focus on optimizing coating thickness, evaluating long-term mechanical behavior under physiological conditions, and exploring synergistic effects with bioactive agents to fully exploit the potential of ALD-functionalized scaffolds.

## 7. Patents

This research was partially a subject of the granted on 20 October 2025 patent no P.447136 titled “Method for manufacturing multi-layer composite foam”, submitted to the Patent Office of the Republic of Poland.

## Figures and Tables

**Table 1:** The characteristics of the microbiological panel selected for the analysis.

*Microorganism*	*Significance in the Field*
*Klebsiella pneumoniae (Gram-negative bacterium)*	*K. pneumoniae* is a clinically significant, opportunistic Gram-negative pathogen, often implicated in severe hospital-acquired infections, including pneumonia, wound, urinary tract, and bloodstream infections. It is commonly associated with multidrug resistance and biofilm formation on medical devices and scaffolds. Testing against K. pneumoniae demonstrates whether the biomaterial provides protection against highly relevant nosocomial bacteria that commonly colonize medical scaffolds or dressings.
*Staphylococcus aureus (Gram-positive bacterium)*	*S. aureus*, including methicillin-resistant strains (MRSA), is a leading cause of wound infections, implant colonization, osteomyelitis, and sepsis. Its ability to adhere to surfaces and form biofilms makes it a primary target in biomaterial evaluation for medical devices, wound dressings, and tissue engineering scaffolds. Effective inhibition of S. aureus growth is thus one of the most essential requirements for materials intended for contact with biological tissues.
*Aspergillus niger (filamentous fungus)*	*A. niger* represents a model of pathogenic filamentous fungi, frequently causing invasive infections in immunocompromised patients, as well as being a major causative agent of biomaterial- and device-associated fungal colonization. For applications in regenerative medicine, prevention of fungal contamination is critical for safety and material longevity.
*Chateomium globosum (saprophytic mold)*	*C. globosum* is a ubiquitous environmental fungus known for its strong cellulolytic and proteolytic enzymatic activity, often involved in the biodeterioration of biomaterials, textiles, and polymers. Testing susceptibility against C. globosum addresses the long-term resistance of materials to environmental fungal colonization and degradation, relevant in both hospital and ambient settings.

**Table 2:** Rheological properties of solutions selected for testing and photos of the final foams.

	*HA 2%* *2.0–2.2*	*HA 2% 2.0–2.2* *+ 1% Silk*	*HA 2% 2.0–2.2* *+ 2% Elastin*	*HA 2%* *Crosslinked*
***k***	55.959	45.790	42.259	0.840
***n***	0.397	0.430	0.453	0.790
	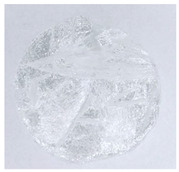	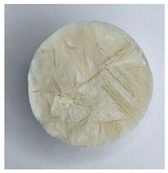	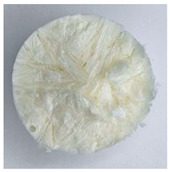	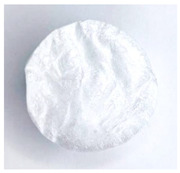

**Table 3:** Thickness of TiO_2_ layer formed after a certain number of cycles (*n*).

*Cycle Numer* *n*	*Layer Thickness [nm]*	*Layer Increment [Å/cycle]*	*Error*	*Correlation R^2^*
*200*	5.7	0.285	0.013	0.19
*600*	16.5	0.275	0.009	0.88
*1200*	31.2	0.260	0.013	0.97

**Table 4:** SEM-EDS analysis of TiO_2_ deposition foams with different number of cycles.

Sample 1—*n* = 200	
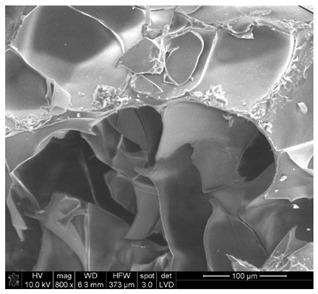	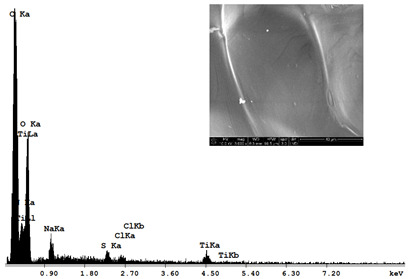
SEM	EDS
Sample 1—*n* = 600	
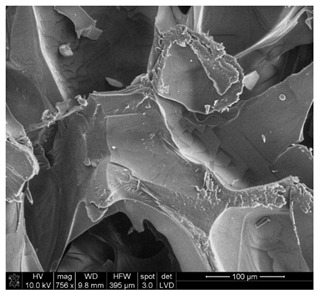	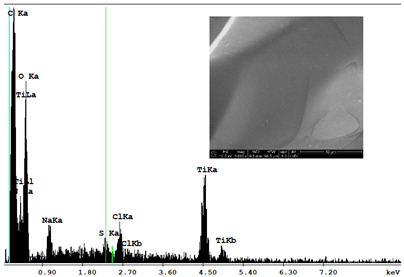
SEM	EDS
Sample 1—*n* = 1200	
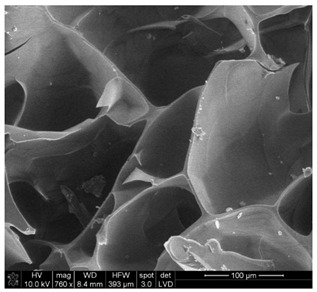	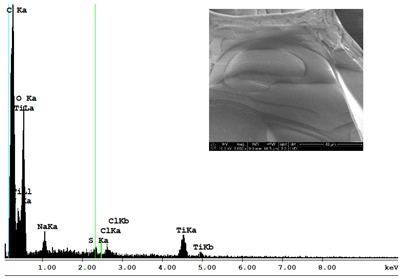
SEM	EDS

**Table 5:** EDS analysis of the quantitative composition (in weighted percentage) of samples after different numbers of cycles.

	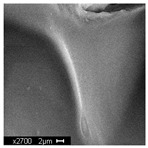	*n* = 200 (%)	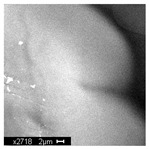	*n* = 600 (%)	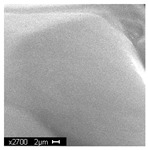	*n* = 1200 (%)
C	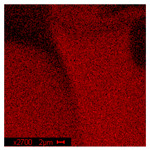	54.38	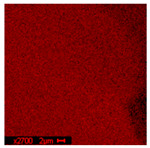	45.74	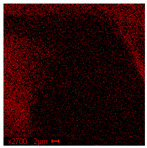	43.00
O	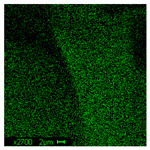	41.01	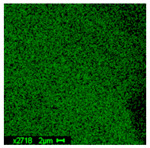	38.88	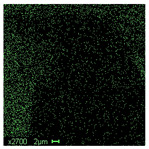	36.02
Na	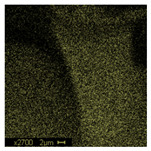	1.99	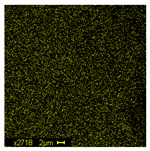	2.13	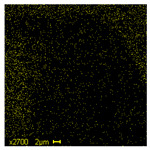	1.64
S	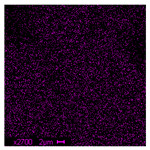	0.53	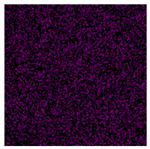	0.68	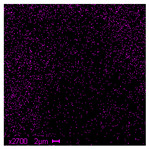	1.25
Cl	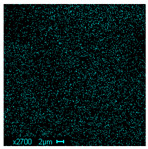	0.41	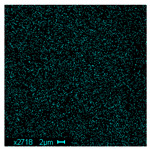	1.67	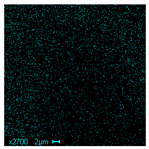	1.78
Ti	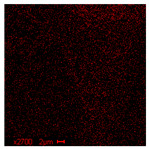	1.64	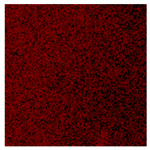	10.89	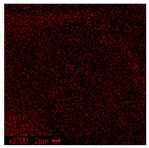	16.30

**Table 6:** The 3D visualizations of internal foams’ structure and 2D visualizations of the edges.

	3D Visualizations		2D Visualizations of the Edges
Sample 1-600	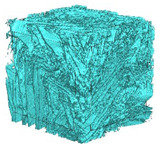	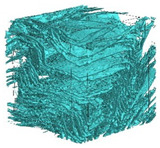	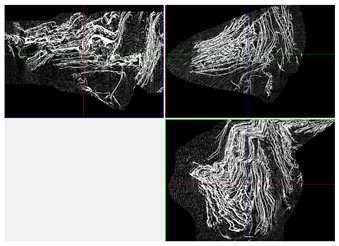
Sample 2-600	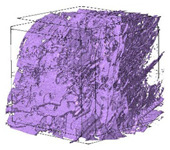	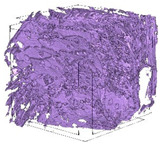	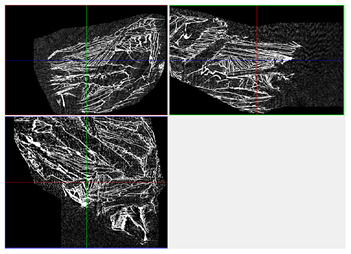
Sample 3-600	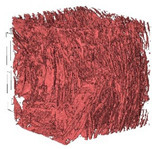	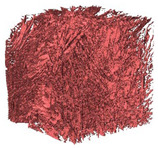	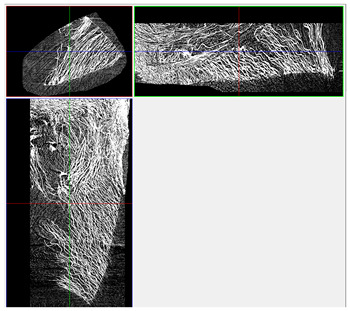
Sample 4-600	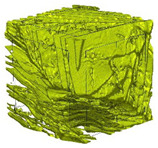	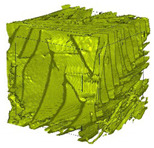	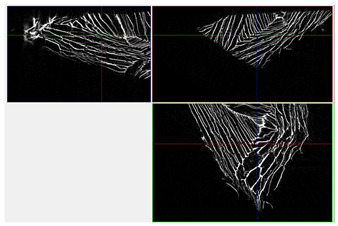

**Table 7:** Comparative analysis of wettability test results for selected samples.

*Sample*	*Composition*	*n = 0* *[px^2^]*	*n = 200* *[px^2^]*	*n = 600* *[px^2^]*	*n = 1200* *[px^2^]*	*Δ200* vs. *0 [%]*	*Δ600* vs. *0 [%]*	*Δ1200* vs. *0 [%]*	*Relative to Sample 1 at n = 0*	*Relative to Sample 1 at n = 1200*
*1*	HA 2%	14.39	13.86	12.58	12.06	−3.7%	−12.6%	−16.2%	—	—
*2*	HA 2% + 1% silk	14.09	13.58	13.02	12.59	−3.6%	−7.6%	−10.6%	−2.1%	+4.4%
*3*	HA 2% + 2% elastin	19.76	19.65	19.51	18.59	−0.6%	−1.3%	−5.9%	+37.3%	+54.2%
*4*	HA 2% crosslinked	28.24	28.03	27.85	24.02	−0.7%	−1.4%	−15.0%	+96.3%	+99.3%

**Table 8:** Solubility test results (sample 1).

	*n = 0 Cycles*		*n = 200 Cycles*		*n = 600 Cycles*		*n = 1200 Cycles*	
t = 0	The foam retains its structural integrity and original dimensions.	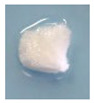	The foam retains its structural integrity and original dimensions.	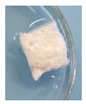	The foam retains its structural integrity and original dimensions.	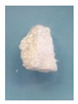	The foam retains its structural integrity and original dimensions.	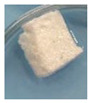
3 days	The foam exhibited a noticeable reduction in volume, accompanied by visible signs of dissolution along its edges. Additionally, an increase in the material’s transparency was observed.		No changes					
9 days	Progression of changes		No changes					
14 days	The foam lost most of its volume, with visible signs of dissolution; the sample transitioned into a hydrogel without a discernible structure.	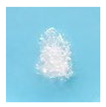	No changes	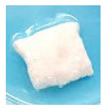	No changes	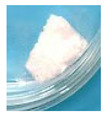	No changes	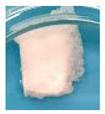

**Table 9:** The microbiological analysis (sample 1) *.

*Sample*	*Klebsiella pneumoniae*	*Staphylococcus aureus*	*Aspergillus niger*	*Chateomium globosum*
*n = 0*	+++	+++	+++	+++
	Strong microbial growth. No inhibition zone was detected, and no reduction in growth was noted compared to the control.	Strong microbial growth. No inhibition zone compared to the control.	Extensive growth, covering the entire surface, with intensity comparable to the control.	Strong microbial growth with intensity similar to the control.
*n = 1200*	-	-	+++	–
	No microbial growth exhibited a clear inhibition zone exceeding 1 mm.	No microbial growth exhibited a clear inhibition zone exceeding 1 mm.	Growth visible without magnification devices, covering up to 25% of the examined area.	No visible growth assessed under a microscope (×50).

* Control material consisted of untreated cotton fabric without any antibacterial finishing. The concentration of the bacterial inoculum (bacterial suspension) was determined to be 2.8 × 10^8^ CFU/mL, representing the number of viable bacterial cells. * Legend: –: No microbial growth observed; clear inhibition zone present. = +++: Strong microbial growth; extensive coverage comparable to the control.

## Data Availability

The original contributions presented in this study are included in the article. Further inquiries can be directed to the corresponding author.
